# A Dual Role of Graphene Oxide Sheet Deposition on Titanate Nanowire Scaffolds for Osteo-implantation: Mechanical Hardener and Surface Activity Regulator

**DOI:** 10.1038/srep18266

**Published:** 2015-12-21

**Authors:** Wenjun Dong, Lijuan Hou, Tingting Li, Ziqiang Gong, Huandi Huang, Ge Wang, Xiaobo Chen, Xiaoyun Li

**Affiliations:** 1 Center for Nanoscience and Nanotechnology, Zhejiang Sci-Tech University, Hangzhou 310018, PR China; 2 School of Materials Science and Engineering, University of Science and Technology Beijing, Beijing 100083, PR China; 3 Department of Materials Science and Engineering, Monash University, Clayton, VIC. 3800, Australia

## Abstract

Scaffold biomaterials with open pores and channels are favourable for cell growth and tissue regeneration, however the inherent poor mechanical strength and low surface activity limit their applications as load-bearing bone grafts with satisfactory osseointegration. In this study, macro-porous graphene oxide (GO) modified titanate nanowire scaffolds with desirable surface chemistry and tunable mechanical properties were prepared through a simple hydrothermal process followed by electrochemical deposition of GO nanosheets. The interconnected and porous structure of the GO/titanate nanowire scaffolds provides a large surface area for cellular attachment and migration and displays a high compressive strength of approximately 81.1 MPa and a tunable Young’s modulus over the range of 12.4–41.0 GPa, which satisfies site-specific requirements for implantation. Surface chemistry of the scaffolds was modulated by the introduction of GO, which endows the scaffolds flexibility in attaching and patterning bioactive groups (such as -OH, -COOH and -NH_2_). *In vitro* cell culture tests suggest that the GO/titanate nanowire scaffolds act as a promising biomaterial candidate, in particular the one terminated with -OH groups, which demonstrates improved cell viability, and proliferation, differentiation and osteogenic activities.

Considering the ageing population, and the giant number of osteoporosis and traffic accident victims, it is a pressing need to design, develop and commercialize synthetic materials for bone repair and replacement, in particular at heavy load bearing sites, such as hip and joints^1^,^2^. The gold criteria in terms of design of manmade biomaterial grafts, emphasize the compatibility with a high degree to those of natural bone tissues, including structure, morphology, topography, chemistry, mechanical properties and biological functionalities^3^,^4^,^5^. Of all existing and being developed biomaterials, scaffolds are the most favourable components for tissue regeneration and replacement, owing to their intriguing structural characteristics, which elicit resemble mechanical performances and functionalities to those of the human bones for satisfactory host-implant interactions, osteoconductivity, and integration^6^,^7^. The porous structure of tissue-engineering scaffolds provides cells with essential space as residence, facilitates exchange of nutrients with metabolic wastes between internal and external environments efficiently^8^, and promotes tissue ingrowth and regeneration^9^,^10^. Meanwhile, adequate mechanical strength is essential to establish a favourable stress environment for the growth of neo-tissues^11^, providing sustainable supports, resisting cell contractile forces and minimizing wear and shrinkage, both *in vitro* and *in vivo*^12^. Matching the mechanical properties well to the site-specific requirements of scaffolds will promote engineering techniques for high yields of robust tissues^13^.

The high porosity (up to 95%) of the synthetic scaffolds gives rise to both enhanced cellular activities and deteriorated mechanical strength^14^. Regarding to the clinical applications, a sound compromise between mechanical integrity and porous structure must be achieved to meet the needs for positional stability, durability, and sustainable integration, which, however, remains a challenge for the development of load-bearing scaffolds for tissue engineering^15^,^16^. Ceramic scaffolds outperform metallic and polymeric counterparts owning to their closer chemical and biological properties to those of natural bone^5^,^7^. An inherent flaw existing in ceramics scaffolds, is their poor mechanical performance. For instance, Young’s modulus, a key index of mechanical properties, of most ceramic scaffolds varies from 10 to 30 MPa^17^,^18^, which is far from the minimum requirement for practical use as structural bone grafts. Such mismatch in the mechanical properties between scaffolds and the host tissues is highly likely to jeopardize the clinical success of the surgical implantation^19^. As such, it is an urgent need to achieve a porous bone graft with a combination of appropriate mechanical properties and osseofunctionalities.

Carbon nanomaterials (fullerene, carbon nanotubes and graphene)^20^,^21^,^22^ have been extensively explored as implant and device candidates for various biomedical and tissue engineering, owing to their excellent biocompatibility^23^,^24^, mechanical and physicochemical properties^25^ .Graphene oxide (GO) with a higher high mechanical strength and the capability of carrying functional groups than that of hydroxyapatite, in particular, has been incorporated into a variety of scaffolds to reinforce their mechanical strength and introduce desirable functionalities^26^,^27^,^28^. Furthermore, endocytic mechanism of GO nanosheets in osteoblasts confirms that both macropinocytosis and microtubules in osteoblasts could effect the general internalization process^29^. For example, scaffolds of chitosan-graphene were prepared via linking the carboxyl groups of GO with the amine groups of chitosan, which facilitated cell attachment and proliferation and improved the stability against enzymatic degradation^30^,^31^. Zhang *et al.* prepared GO/PVA composite hydrogels using a freeze/thaw method and the mechanical properties of the hydrogels were significantly improved^28^.

Titanate/titania is also a key biomaterial candidate with excellent biocompatibility, improved *in vitro* cellular responses, accelerated *in vivo* osseointegration and outstanding drug delivery behaviours^32^,^33^,^34^. Titanate based scaffolds were prepared from titanium substrate through a simple hydrothermal treatment, demonstrating promoted cell adhesion and proliferation, controlled on-site drug release, and photocatalytic sterilization^32^. Addition of nano hydroxyapatite particles into titanate scaffolds leads to elevated *in vitro* stability and bioactivity, however, it remains unclear what correlation between the addition of hydroxyapatite and mechanical functions of the scaffolds^33^. In addition, surface-active groups that are sensitive to a variety of bioactive molecules, play a profound role in regulating bioactivity of the scaffolds^35^,^36^. In general, a scaffold grafted with bioactive molecules is active to integrins through covalent bonding and presents a great cellular affinity^37^, which dominates cell adhesion strength, regulates proliferation, migration and differentiation^38^, and modulates the signal transduction, such as growth factors and cytokines^39^.

Herein, we incorporated GO sheets into a hydrothermally prepared porous titanate nanowire scaffold through an electrophoretic deposition approach to optimize the mechanical properties of a porous titanate nanowire scaffold and introduce new surface chemistry to the titanate scaffolds via various functional groups linked with GO sheets. We hypothesize that such GO sheet inclusions perform dual functions as strength hardener and bioactivity stimulator in the resulting titanate scaffolds. To validate this, microstructure, representative mechanical properties and bioactivity of the GO modified titanate scaffolds were characterised. Results reveal the GO modified titanate scaffolds demonstrate an interconnected porous structure, a flexible and bioactive interface, and a satisfactory mechanical strength. The compressive strength approaches approximately 81.1 MPa and Young’s modulus varies over a range of 12.4 GPa to 41.0 GPa, which satisfy site-specific requirements for implantation. Surface chemistry of the modified scaffolds induces flexibility in attaching and patterning bioactive groups (i.e. -OH, -COOH and -NH_2_), and thus enhances cellular affinity. Apart from polymeric biomaterials, the porous feature of the GO/titanate scaffolds originated from the surface of Ti or Ti alloy implants can provide a unique 3D environment containing the pathways and channels to facilitate vascularization. *In vitro* cell culture tests discover that the GO/titanate scaffolds, in particular the one functionalized with -OH groups, exhibit a profound strength to improve in cell viability, proliferation, differentiation and osteogenic activity. It is evident that the biocompatible GO/titanate scaffolds can retain their structural integrity and provide sufficient biomechanical supports for tissue regeneration even bearing constant loads.

## Results

### Characterization of GO/Titanate Nanowire Scaffolds

**Figure 1:**
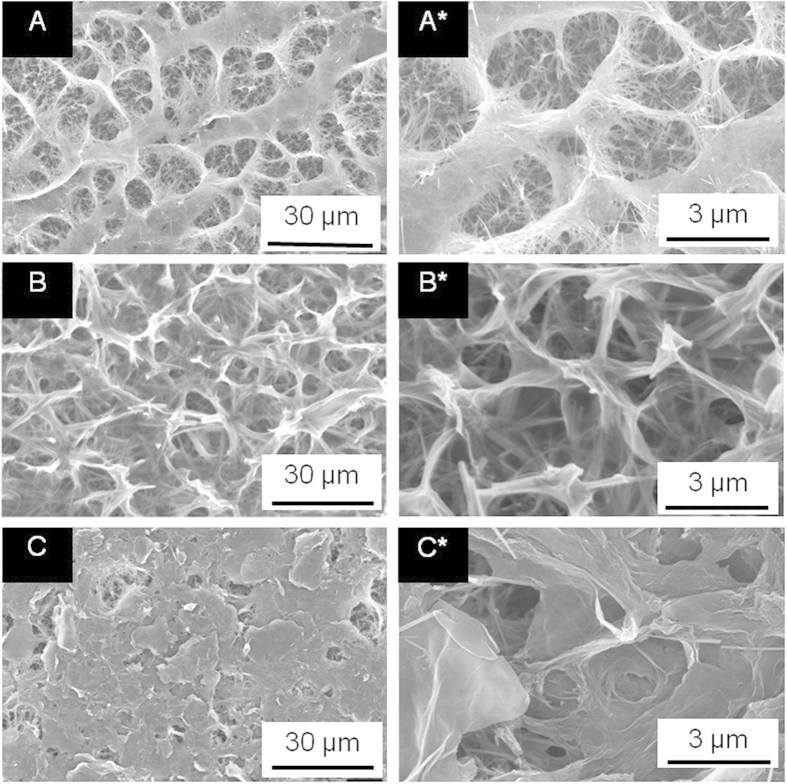
Surface morphology of GO modified titanate nanowire scaffolds experiencing different processing time durations of electrodepositon: (A and A*) 2 min, (B and B*) 5 min and (C and C*) 10 min, respectively. (Electrodeposition condition: pH = 8.5 and Eappl = 1.3 V_SCE_). It is evident that the quantity and density of the GO deposition on titanate scaffolds increases as a function of processing time.

The formation of titanate nanowire scaffolds on the surface of Ti substrate through hydrothermal process, which are usually described as titanium hydroxide oxide [Ti_3_(OH)_2_O_5_]^43^, was confirmed by SEM and HRTEM micrographs and XRD pattern (see Supplementary Fig. S4 online). The original titanate nanowire matrix with characteristic interconnected macropores of 2–10 μm in diameter (see Supplementary Fig. S4A online). XRD result also confirmed that GO maintain its original oxide state during the electrophoretic deposition (see Supplementary Fig. S4D online). The effects of deposition processing time, temperature and pH of the electrolyte and externally applied current value on the morphology of the GO/titanate nanowire scaffolds were systematically studied. SEM micrographs (Fig. 1) reveal the correlation between electrodeposition time duration and the surface morphology of GO/titanate nanowire scaffolds. A rapid deposition process, i.e. 2 min, led to a bunch of GO sheets randomly scattering over partial surface of the titanate nanowire matrix (Fig. 1A and A*). When intermediate electrochemical deposition time of 5 min was adopted, growth of GO sheets and 3D converge on titanate was significantly stimulated. GO sheets were embedded into pores and covered across the entire bottom surface and walls of the caves. It is evident that GO sheets grew along the contour of mesostructure of the titanate matrix and established a strong epitaxial 3D porous structure (Fig. 1B and B*). When deposition time was prolonged to 10 min, numerous dense GO sheets covered the titanate nanowire scaffold and sealed interconnected porous structure (Fig. 1C and C*). Both the pathways and channels for expected vascularization were blocked, which would inhibit the bone ingrowth through the scaffolds.

pH value of the electrolytes presents a critical impact on the morphology of GO/titanate nanowire scaffolds (E_appl_ = 1.3 V and t = 5 min, see Supplementary Fig S5 online). The most acidic electrolyte (pH 7.2) gave rise to unperceivable deposition of GO sheets after 5 min deposition at 1.3 V (see Supplementary Fig. S5A and A* online). A slight increase in pH value to 7.6 elicited a uniform growth of GO sheets with a low density (see Supplementary Fig. S5B and B* online). Further increasing pH value to 8.5, facilitated the formation of GO sheets to cover across the entire bottom surface and walls of the caves and establish a strong epitaxial 3D porous structure (see Supplementary Fig. S5C and C* online). In addition, externally applied potential contributes greatly to the morphology of the GO/titanate nanowire scaffolds (see Supplementary Fig. S6 online). It can conclude that 1.3 V is the premium condition to generate GO sheet deposition with uniform distribution and sufficient quantity and density to perform desired function. The optimal GO deposition on titanate nanowire scaffolds needs to be carried out at 1.3 V applied potential, in pH 8.5 electrolyte for 5 min. According to the precedent structural characterization, GO/titanate nanowire scaffolds with desirable macroporous and interconnected structure were fabricated (Fig. 1B and B*). As such, the GO/titanate nanowire scaffolds inheriting the mesostructure from titanate nanowire matrix and constructing a strong epitaxial 3D porous structure, enlarged interfacial area and aspect ratio, are desirable for osteoblast to adhere onto the material surface, which will be evaluated in the following.

### Young’s Modulus and Compressive Strength of GO/Titanate Scaffolds

**Figure 2:**
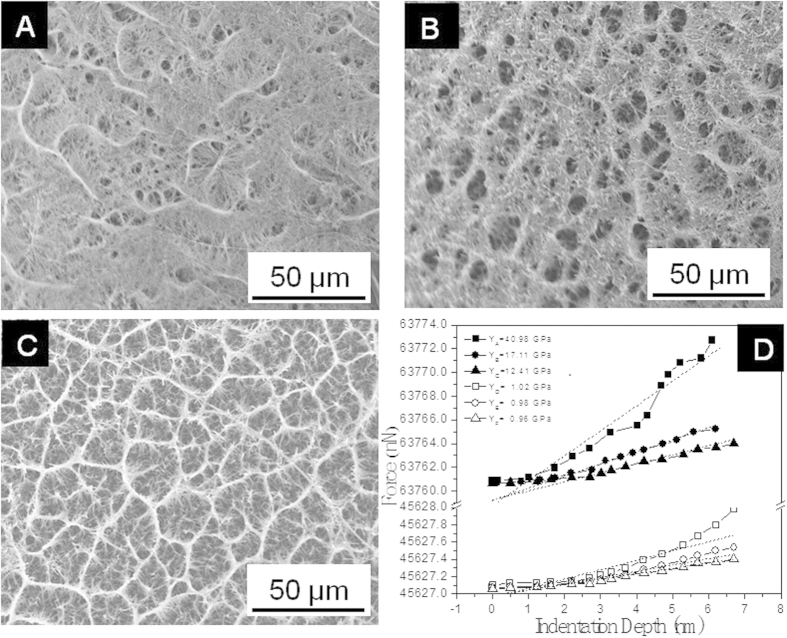
SEM images of the titanate nanowire scaffold obtained by hydrothermal method under 240 °C for (**A**) 4 h, (**B**) 6 h, (**C**) 8 h, followed by electrodeposition of GO sheets at identical conditions (i.e. E = 1.3 V_SCE_, pH = 8.5 and t = 5 min), and (**D**) evaluation of Young’s modulus of GO modified titanate nanowire scaffolds (Y_A_, 4 h; Y_B_, 6 h and Y_C_ 8 h), in comparison with that of neat titanate scaffolds (Y_D_, 4 h; Y_E_, 6 h and Y_F_, 8 h). The relationship of sample deflection to the applied force was analyzed with aid of the FMA software. It suggests that the introduction of GO has a significant impact (over 10 folds increment) on the elasticity of the titanate scaffolds.

To validate the hypothesis that inclusion of GO sheets can improve mechanical properties of the parent titanate scaffolds, we prepared three typical scaffolds (Fig. 2A–C), obtained via hydrothermal process for discrete time durations, i.e. 4, 6 and 8 h, respectively, and then modified by GO under the abovementioned optimal processing conditions (i.e. E = 1.3 V, pH = 8.5 and t = 5 min), for mechanical property analysis in the form of force - indentation depth curve containing a slope as the index of Young’s modulus. Young’s modulus of the respective titanate nanowire scaffolds hydrothermally processed for 4, 6 and 8 h are 1.02 (Y_D_), 0.98 (Y_E_) and 0.96 GPa (Y_F_), whilst that of the GO modified scaffolds was more than one order of magnitude higher (40.98 (Y_A_), 17.11 (Y_B_) and 12.41 GPa (Y_C_)), suggesting a profound strengthening stimulus of GO sheets. It is also noteworthy that the curve proceeds steeper with reduction in hydrothermal reaction time. Moreover, indentation depth was detected when the force increased to 67.6 μN ± 0.03 μN for the three groups of GO modified scaffolds, corresponding to compressive strength of approximately 81.1 MPa and suggesting a superior compressive strength. Meanwhile, the mechanical properties of the functionalized GO/titanate nanowire scaffolds are close to the non-functionalized GO/titanate counterparts after hydrothermal process for 4 h, as such the presence of these functional groups demonstrate marginal impact on the yielded mechanical properties (see Supplementary Fig. S7).

### Surface Chemistry Alteration through Functionalization of GO/Titanate Scaffolds

**Figure 3:**
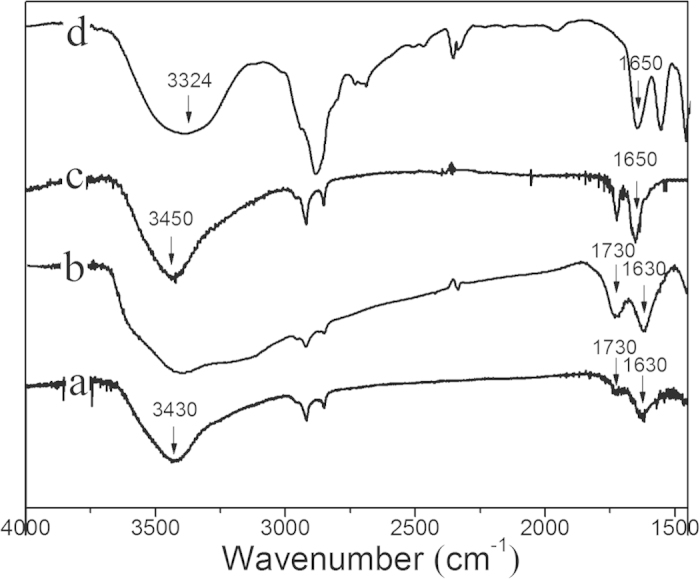
FTIR-ATR analysis of (**a**) GO/titanate scaffold, (**b**) -COOH grafted GO/titanate scaffold, (**c**) -OH grafted GO/titanate scaffold and (**d**) -NH_2_ grated GO/titanate scaffolds, analyzed in the 1500 cm^−1^ ~ 4000 cm^−1^ region.

In addition to expected mechanical strengthening performance, we also introduced GO sheets into titanate scaffolds pursuing optimization of surface chemistry for a cell-favourable interface. Towards this specific surface chemistry, -COOH, -OH and -NH_2_ terminals were constructed through covalent bonding and the structural evolution from GO to GO-COOH, GO-OH and GO-NH_2_. The FTIR-ATR pattern of the GO sheets (Fig. 3a) reveals the presence of oxygen-containing functional groups, including -COOH and -OH. These functional groups made the GO highly hydrophilic and dispersible. When GO were activated with -COOH, some -OH groups were sacrificed during the strong alkaline condition, intensity of the absorption peak around 3430 cm^−1^ (from -OH stretching mode) becomes lower. The peaks at 1730 and 1630 cm^−1^ are characteristics of grafted -COOH (Fig. 3b)^44^. When Tris or propane diamine was further grafted to GO through covalent bonding, peak shifting to 1650 cm^−1^ of the characteristic amide-carbonyl (NH-CO) stretching vibration (Fig. 3c and d), indicates the reaction of -NH_2_ groups with grafted -COOH groups in the presence of EDC^45^. Moreover, the broad peak at 3450 cm^−1^ originating from -OH stretching vibration (Fig. 3c) proves the combination of -OH groups with GO sheets^46^. It is apparent that -NH_2_ groups grafted on GO as absorption peaks for N-H stretching vibration at 3324 cm^−1^ and the N-H bending vibration at 1650 cm^−1^ (Fig. 3d)^47^.

### Osteoblast Adhesion, Spreading, Proliferation and Cytotoxicity of the Functionalized Scaffolds

**Figure 4:**
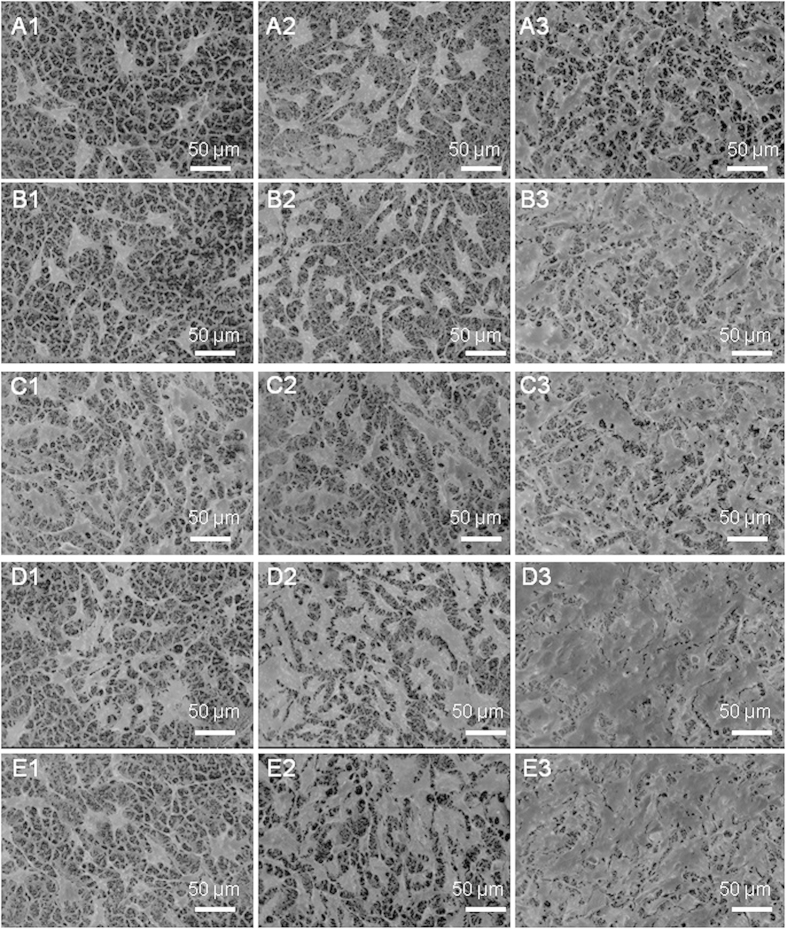
SEM micrographs of the attachment of MG63 cells on (**A**) neat titanate scaffold, (**B**) GO/titanate scaffold, (**C**) -COOH grafted GO/titanate scaffold, (**D**) -OH grafted GO/titanate scaffold and (E) -NH_2_ grafted GO/titanate scaffold for 2, 4, 6 days. The numbers of 1–3 are designated to the culture time duration of 2, 4 and 6 days, respectively.

**Figure 5:**
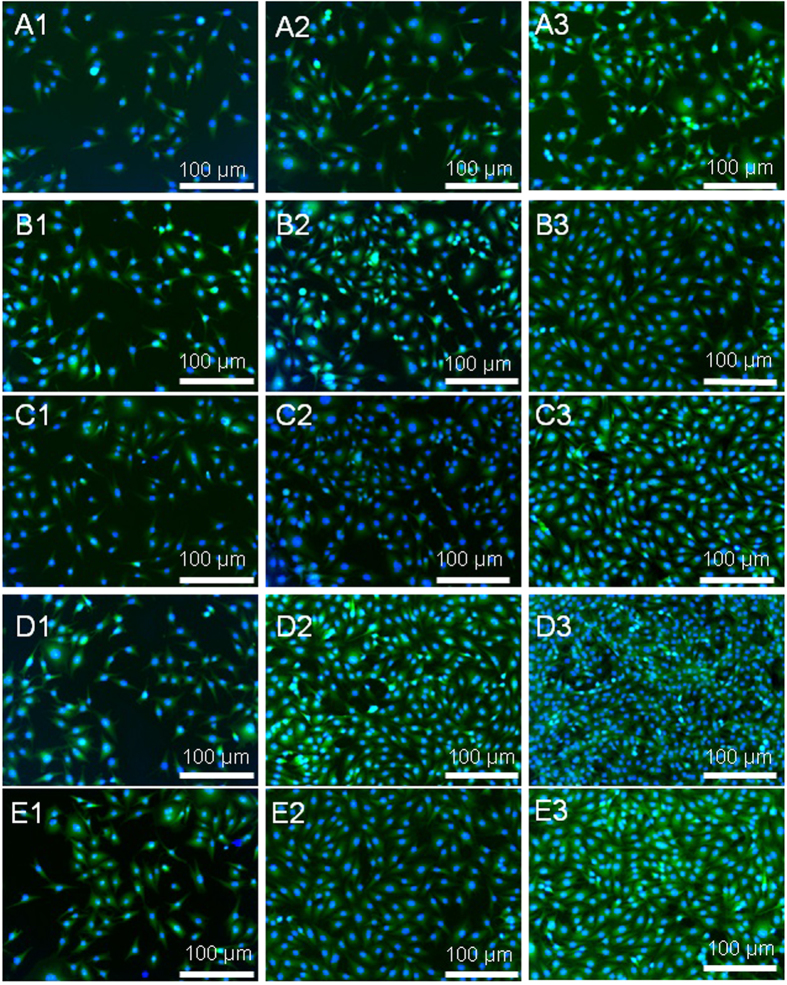
Fluorescence microscopy of attachment of MG63 cells (green, labeled with phalloidin-FITC, counterstained with DAPI for unclei in blue) on (**A**) neat titanate scaffold, (**B**) GO/titanate scaffold, (**C**) -COOH grafted GO/titanate scaffold, (**D**) -OH grafted GO/titanate scaffold and (**E**) -NH_2_ grafted GO/titanate scaffold for 2, 4, 6 days. The numbers of 1–3 are designated to the culture time duration of 2, 4 and 6 days, respectively.

In general, cell attachment, which highly correlates to surface chemistry, dominates tissue-engineering progress in particular at initial stage, because cell attachment occurs promptly upon in contact with scaffolds and therefore governs subsequent activities, such as cell spreading, migration, differentiation and eventually extracellular matrix formation. Though enhanced cellular attachment of MG63 cells after 2, 4 and 6 days’ culture to the surface of the functionalized scaffolds was identified from SEM micrographs (Fig. 4), the degree of such improvement in cellular attachment is highly dependent on the surface chemistry of the scaffolds tested and culture time duration. At the relatively onset culture stage, i.e. day 2, the -COOH functionalized scaffolds illustrate the most favorable cell adhesion of all scaffold groups, whilst the scaffolds (neat titanate and GO/titanate) without any functional groups provide a surface with the least attraction to cells. At the intermediate culture stage, i.e. day 4, the scaffold combined with -OH group terminals presents the highest density of attached cells, in the contrary, the lowest cell density was observed on the neat titanate scaffold. The longest culture time (6 days) facilitates osteoblasts attachment and proliferation, which yields cell clusters on the -OH and –NH_2_ functioned scaffolds. The fluorescence microscopy images of live cells on the 5 scaffolds (Fig. 5) unveil that the live cells on the scaffolds appear as viridity spots and related cytoblasts as purplish blue dots. The -OH functionalized GO/titanate nanowire scaffold provides MG63 cells a superior adhesion and bioactivity than the others, which is in agreement with the SEM observation.

Surface roughness is of paramount importance in the design and manufacture of implantable biomedical devices. Particularly in the case of bone implants, roughness has been introduced in order to influence bone ingrowth and early stabilization. In general, rough scaffolds could provide larger surface areas to interact with protein molecules than that of dense components, which are beneficial for cell adhesion. Surface roughness of the scaffolds was analyzed to elucidate its engagement in cellular behaviours (see Supplementary Fig. S8 online). R_a_ value of the GO/ titanate scaffolds (1.94 μm) is close to that of the neat titanate scaffolds (1.80 μm). Addition of functional group terminals introduces a slight increase in R_a_ value of the GO/ titanate scaffolds (-COOH, 2.15; -OH, 2.26; and –NH_2_, 2.16 μm). Furthermore, the adsorption of protein BSA to the surface of the scaffolds after functionalization using -COOH, -OH or -NH_2_ groups was promoted dramatically. In particular, the -OH modified GO/titanate nanowire scaffold presents the most outstanding capability to boost protein adsorption (see Supplementary Fig. S9 online).

**Figure 6:**
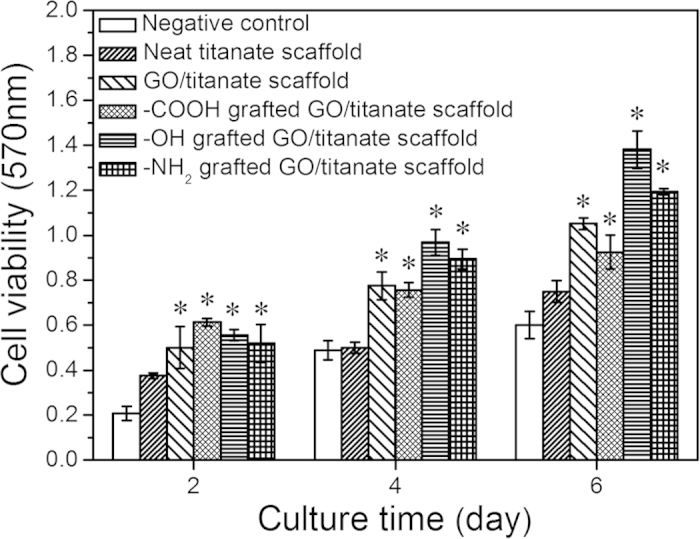
Comparison of the proliferation of MG63 cells on neat titanate scaffold, GO/titanate scaffold, -COOH grafted GO/titanate scaffold, -OH grafted GO/titanate scaffold, and -NH_2_ grafted GO/titanate scaffold, i.e. 2, 4 and 6 days, respectively. Negative control refers to the incubation of cells with the medium. Positive control represents the incubation of cells with the 1% triton containing medium. (*Significant against the MTT assay on neat titanate scaffold at p < 0.05, and error bars represent standard deviation).

The cytotoxicity and behaviors of GO in biological system are of paramount importance^48^, which is a major criterion for an ideal tissue engineering scaffold, i.e. a material should be nontoxic to the stem or progenitor cells that are recruited to the injured site. The number of the living cells cultured on the surface of various scaffolds was reflected by MTT assays (Fig. 6) and results reveal that introduction of GO sheets into titanate scaffolds and grafting of -OH, -COOH and -NH_2_ groups accelerate cellular proliferation without any evidence of cytotoxicity. A higher number of osteoblasts were found on the GO/titanate nanowire scaffold and its functionalized counterparts than that on the controls (p < 0.05). It is intriguing to see that the -COOH modified scaffold presents the highest OD value after 2 days’ culture, whilst an opposite phenomenon was observed after 4 days’ culture. The increment in viable cells on -OH and -NH_2_ modifying scaffolds exceeds that on the -COOH modified scaffold. After 6 days’ cell culture, the highest cell density of viable cells is detected on the scaffold with -OH terminals of all scaffolds. Notably, the GO/titanate nanowire scaffold with -OH group terminals demonstrates a most favourable interfacial environment for MG63 osteoblasts’ proliferation and differentiation.

**Figure 7:**
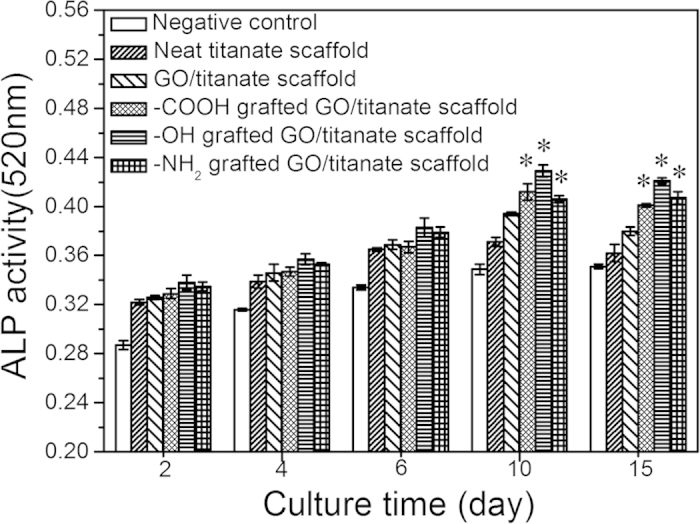
ALP activity of MG63 cells on neat titanate scaffold, GO/titanate scaffold, -COOH grafted GO/titanate scaffold, -OH grafted GO/titanate scaffold, and -NH_2_ grafted GO/titanate scaffold, i.e. 2, 4, 6, 10 and 15 days. Negative control refers to the incubation of MG63 cells with medium. Positive control demonstrates the incubation of cells with 1% triton containing medium. (*Significant against ALP activity on neat titanate scaffold at p < 0.05, and error bars represents the standard deviation).

Early osteogenic differentiation, bone formation and matrix mineralization were evaluated by ALP activity analysis (Fig. 7). In general, the formation of multilayered clusters indicates increased ALP expression and the mineralization. It is apparent that the -OH, -COOH, -NH_2_ functionalized scaffolds elevate ALP enzymatic activities. In particular, the ALP contents of the cells cultured on the GO/titanate nanowire scaffolds were significantly higher than that on the neat titanate counterpart (*p* < 0.05), which suggests an enhanced biocompatibility and bioactivity, and an evident osteogenic induction. Furthermore, -OH modified scaffolds provide favourable nuclei sites for evolution of calcium phosphate crystals^49^,^50^. Cell growth on the -OH modified GO/titanate nanowire scaffold exhibit an higher increment in ALP activity than other functioned GO/titanate nanowire scaffolds, which confirms the up-regulation of ALP enzymatic activity, and matrix mineralization of the -OH terminal scaffolds.

**Figure 8:**
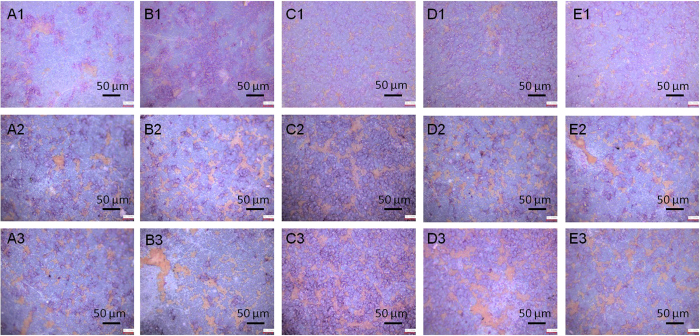
Representative images of ARS assay of MG63 cells on (**A**) neat titanate scaffold, (**B**) GO/titanate scaffold, (**C**) -COOH grafted GO/titanate scaffold, (**D**) -OH grafted GO/titanate scaffold, and (**E**) -NH_2_ grafted GO/titanate scaffold for various culture time durations, i.e. 2, 6 and 15 days, which are represented by numbers 1, 2 and 3, respectively.

**Figure 9:**
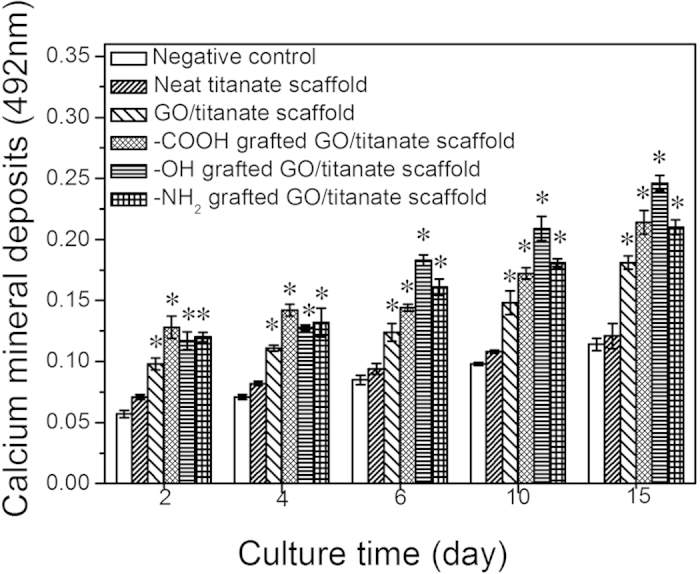
Quantitative measurements of mineral deposition in MG63 cells by Alizarin Red-S staining on neat titanate scaffold, GO/titanate scaffold, -COOH grafted GO/titanate scaffold, -OH grafted GO/titanate scaffold, and -NH_2_ grafted GO/titanate scaffold, i.e. 2, 4, 6, 10 and 15 days, respectively. Negative control refers to the incubation of MG63 cells with medium. Positive control demonstrates the incubation of the cells with 1% triton containing medium. (*Significant against the quantified mineral deposits on neat titanate scaffold at p < 0.05, and error bars represent standard deviation).

ARS staining was used to characterize the bone nodule formation of the MG63 cells cultured on different scaffolds for 2, 6, 15 days (Fig. 8). The orange bright stains on the scaffolds are the sign of calcium deposition. Therefore, the presence of more positive and brighter orange stains on various GO/titanate scaffolds than that on the neat titanate nanowire scaffold indicates a higher calcium deposition. The results of quantitative analysis (*p* < 0.05) depicted in Fig. 9 indicate that the functionalized scaffolds present negative surfaces in cell culture medium containing serum proteins, especially the -COOH containing scaffold demonstrates the highest ζ-potential than those of the -OH and -NH_2_ grafted scaffolds. Stronger negative surfaces elicit a greater inductive capability for heterogeneous nucleation and growth of hydroxyapatite crystals. As such, the calcium deposition on -COOH functioned scaffold is significantly higher than that on other scaffolds after 2 and 4 days’ incubation. And -OH functioned scaffold presents the best calcium deposition afterwards.

## Discussion

Titanate nanowire scaffolds prepared through simple hydrothermal treatment demonstrate an interconnected porous morphology with high resemblance to that of natural bone tissues, which supply a favourable environment to stimulate osseo-activities, such as cell attachment, proliferation, differentiation and extracellular mineralization^31^. Incorporation of nano hydroxyapatite particles onto the titanante scaffolds gives rise to a substantial increment in *in vitro* bioactivity and yields a satisfactory *in vivo* integration between host bone and scaffold^32^. However, the poor mechanical strength, a consequence of porous configurations, still elicits severe concerns about the integrity and service lifespan of the scaffold grafts in clinical applications, in particular for load-bearing fractures^7^.

The results derived from the present study reveal GO sheets play a dual role in regulating mechanical properties and surface chemistry of parent titanate scaffolds, which were fabricated onto titanate wires through electrodeposition. Morphology, density, spatial distribution of the GO sheets on the surface of titanate wires correlate closely to the processing parameters, including time duration, pH of the electrolytes, and externally applied potential. An optimal processing time of 5 min was selected to generate sufficient quantity of GO sheets to introduce desirable functions and avoid excessive deposition to block the open pores (Fig. 1). It is evident that an increase in pH value leads to accumulation of negative charges on GO sheets that migrate towards the positively charged titanate nanowire scaffold as cathode, driven by the electric field force upon applying a DC voltage. Therefore, the resulting density of GO sheets is a function of pH (see Supplementary Fig. S5 online). Furthermore, a high (externally applied) potential is also desired to produce GO sheet deposition with uniform spatial distribution and sufficient quantity and density to play the anticipated performances. The optimal processing parameters are concluded as follows: E_appl_ = 1.3 V, pH = 8.6 and t = 5 min.

It is well-known that Young’s modulus of the human bones vary individually and even different regions of the same bone component display discrete values^51^, normally over the range of 10–40 GPa^52^. Such high elasticity of bone remains a challenge for numerous synthetic scaffolds except for metallic ones^7^, however the latter is associated with severe concerns about wear and corrosion in physiological environments^52^. Therefore, it is an urgent requirement to find a pathway to optimize the poor mechanical strength of scaffolds to match that of the human bones. It has come up with that 0.8wt.% addition of GO into PVA hydrogel matrix leads to a 132% increase in tensile strength and a 36% improvement of compressive strength^27^. It was found that the elastic modulus and hardness of chitosan can be increased by 44% and 52% due to the presence of 1wt.% GO^29^. Such dramatic contribution to mechanical strengthening is attributed to the large specific surface area and distinctive two-dimensional structure of GO, which is likely to impose a higher degree of geometric constraint with regard to the matrix^53^. It is noteworthy that Young’s modulus of the titanate nanowire scaffolds can be radically elevated (over ten folds) through introducing GO inclusions onto titnate nanowires (Fig. 2D), which can be attributed to the high strength of individual GO sheets^25^,^54^. More encouragingly, by means of tuning pore size of the parent titanate scaffolds and density of GO deposition, a broad range of Young’s modulus can be achieved to match those of the tissues at the site of implantation (Fig. 2). Ongoing work of our group is pursuing this fascinating strengthening methodology to scaffolds with larger pore size (50–100 μm) and higher porosity (up to 90%) that are the advocated settings associated with successful tissue regeneration at load-bearing sites^7^, where adequate toughness and stiffness are also essential.

Surface chemistry is one of the dominant factors to regulate cellular responses to the implanted scaffolds^55^. Protein adsorption and cell attachment are the very first interactions upon the surface of implanted scaffolds, which modulate the subsequent cell proliferation, differentiation and mineralization^56^. As such, surface chemistry of the titanate scaffolds was modified through GO sheets bearing discrete functional terminal groups, i.e. -COOH, -OH and -NH_2_. At the initial culture stage, i.e. 2 days, a larger number of cells attached on the -COOH functionalize scaffold is attributed to the high affinity to both α_5_β_1_ and α_V_β_3_ integrins of -COOH terminals, compared with -OH and NH_2_ terminated surfaces which are active to α_5_β_1_ integrins exclusively^57^. Given that oxygen-containing groups on the GO surface behave as bioactive molecules and provide numerous active sites for integrins and cell attachment, a significant difference in cell density was observed between the surfaces of GO/titanate scaffolds and neat titanate counterparts. After such initial attachment, MG63 cells spread and proliferate on the scaffolds and penetrate into the porous areas with the tentacles elongated into the micro-holes. At the intermediate culture stage, i.e. day 4, the scaffold combined with -OH group terminals presents the highest density of attached cells, in the contrary, the lower cell density was observed on the neat titanate scaffold, indicating the promotion of osteogenesis by the modification derived from addition of GOs and further enhancement through the functionalization of -OH groups, whereas the -COOH functioned scaffold, with significant ανβ3 integrin binding, inhibits osteoplastic differentiation^58^. On the other hand, the surface terminated by -COOH groups, having a high ζ-potential in cell culture medium containing serum proteins, could donate electrons to oxygen molecules and induce reactive oxygen species (ROS), and then lead to disorder of mitochondrial functions and the cell death^59^. The results of all biological assays illustrate the scaffold terminated with -OH groups demonstrated improved cell viability, and accelerated proliferation, differentiation and osteogenic activities accordingly, which can be attributed to ease of modifying such particular surface to attach growth factors, adhesion proteins, or other molecules of biological importance which dominate adsorption kinetics, cell adhesion strength, and even focal adhesion assembly and signalling. Given the selective binding of α_5_β_1_ integrin to -OH, such surface-dependent differences in integrin adsorption have a profound influence on cellular activities, including integrin receptor binding and subsequent cell adhesive events. The scaffold grafted with -OH also provides favorable sites for calcium phosphate nucleation and supports higher recruitment levels of proteins for cytoskeleton and signalling^60^. In addition, binding GO sheets with various functional terminals to introduce radical alteration on surface chemistry, exhibit negligible impact on the mechanical properties of the parent scaffold (see Supplementary Fig. S7 online), which provides a feasible pathway to yield desirable surface nature for orthopaedic applications.

## Methods

### Preparation of Titanate Nanowire Scaffolds

The titanate nanowire scaffold was prepared through a hydrothermal process as described previously^33^. Briefly, titanium plates with a dimension of 10 mm × 10 mm × 0.2 mm were hydrothermally treated in a sealed Teflon-lined vessel containing 20 mL of 1.0 mol/L NaOH solution at 240 °C for 8 h, followed by ultrasonic cleaning in 10 mL of acetone and absolute ethanol for 15 min at room temperature, respectively, rinsing with deionized water and dried in air thereafter.

### GO Synthesis and Its Carboxylation, Hydroxylation and Amination

Graphitic oxide was prepared from natural graphite by modified Hummers method^40^,^41^. The yielded mixture was washed by repetitive centrifuging and filtration with 5% HCl aqueous solution and then deionized water. 160 mL water was added to the final product and vortexed well to make an even suspension for further use. Then GO-COOH was prepared using sodium chloroacetate (Cl–CH_2_-COONa) in a strong basic solution to activate epoxide and ester groups, and to convert the –OH to –COOH. In brief, 5 mL of the prepared GO aqueous suspension was diluted into 10 mL solution, and then bath sonicated for 1 h to make a clear solution. 1.2 g NaOH was added into the 10 mL GO suspension (~2 mg/mL) and bath sonicated for 2 h followed by addition of 1.0 g sodium chloroacetate (Cl-CH_2_-COONa) and sonicated for another 3 h to convert the -OH groups to -COOH via conjugation of acetic acid moieties (designated as GO-COOH hereafter).

Regarding to the hydroxylation and amination processes, the obtained GO-COOH solution was neutralized, and purified by repetitive rinsing and filtrations. Tris (aladin Inc.) and propane diamine were added into the GO-COOH suspension, respectively and sonicated for 30 min to make a homogeneous mixture. N-(3-Dimethylaminopropyl)-N’-ethylcarbodiimide (EDC, from aladin Inc.) was added twice every 4 h to activate the reaction of -COOH and -NH_2_. The final products were yielded after repetitive centrifuging and filtrations.

### Titanate Nanowire Scaffolds Incorporated with GO Sheets

The deposition of GO nanosheets on titanate nanowire scaffolds were carried out on DYY-6D electrophoretic instrument consisting a home-built two-electrode cell containing 100 mL of 0.02 mg/mL GO nanosheets suspension, where titanate nanowire scaffolds grew on Ti plates were utilized as working electrode (WE), a platinum plate as counter electrode (CE), and commercial saturated calomel electrode (SCE) as reference electrode (see Supplementary Fig. S1 online). The deposition process was monitored using a SCE with a potential range from 1.0 to 1.3 V_SCE_, and the current (voltage) was in the range of 0.1 ~ 5 mA (1.0 ~ 10 V_SCE_) at room temperature for 5 ~ 15 min.

### Structural Characterization of GO/Titanate Nanowire Scaffolds

Surface topography of the GO/titanate nanowire scaffold was examined through field emission scanning electron microscopy (FE-SEM, HITACHI S-4800, Japan) and high resolution transmission electron microscopy (HRTEM, JEM-2100F operated at 200 kV). Gold-palladium coating (~10 nm) was deposited prior to EM observation. Surface roughness of the scaffolds was measured on a confocal laser scanning microscopy (CLSM; KEYENCE, Japan). Six replicates of surface roughness measurement were conducted to guarantee reproducibility. Structural and molecular composition of the GO/titanate nanowire scaffolds was evaluated using a Nicolet 5700 Fourier transform infrared spectroscopy-attenuated total reflection spectrometer (FTIR-ATR) with a resolution of 4 cm^−1^ and a scan number of 32, and the spectra were recorded from 1450 to 4000 cm^−1^.

### Young’s Modulus Measurement

Mechanical tests were carried out using micromanipulator (MM3A Kleindiek Nanotechnik) equipped with the FE-SEM. It consists of a tungsten needle mounted on the three-axis micromanipulator and positioned vertically to the surface of the scaffold (schematic illustration in Supplementary Fig. S2 online). FE-SEM micrograph for each load was acquired and analyzed through Force Measurement Analysis (FMA) software to obtain the force-indentation depth curve. The first peak of the deformation curve, often defined as an upper yield stress, was used to calculate compressive strength^42^. Young’s modulus of the scaffolds was determined by the force-indentation depth curve with the micromanipulator driven in its highest spatial resolution. Load was applied and increased by moving the tungsten tip upwards with a series of defined paces (ranging from 1 to 128) towards the sample. For each load, an SEM micrograph was recorded and stored to determine the displacement.

While direct force measurements were possible with shifting the spring table, the indentation depth was determined directly by automated image analysis of the micrographs acquired for each load during the test. After the test, the tip apex diameter of tungsten needle was determined from scratch on aluminum foil, which is 0.5 μm at the innermost region (see Supplementary Fig. S3A online). The thickness of the titanate scaffolds was determined through cross-sectional observation (see Supplementary Fig. S3B online).

Young’s modulus of the scaffolds was determined using standard Hooke’s law:


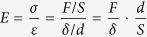


Where *F* is the applied load, *δ* the indentation depth, S the cross-sectional area of the tungsten tip, *d* the thickness of the scaffold, and *F/δ* represents the slope of the force-indentation depth curve in the linear region of loading curve.

### ***In Vitro*** Cell Culture Evaluation

Human MG63 cells, an osteosarcoma cell line (Shanghai Institute of Biochemistry and Cell Biology, Shanghai, China), were cultured in Dulbecco’s Modified Eagles Medium (DMEM) supplemented with 10% fetal bovine serum (FBS) and antibiotics (100 U/ml penicillin, 100 mg/ml streptomycin). MG63 cells were digested using 0.25% trypsin for 3–5 min, centrifuged at 800 g for 5 min and resuspended in the medium. Neat and GO modified titanate nanowire scaffolds were immersed in 24-well culture plate containing 75% alcohol under UV irradiation for 1 h for sterilization. Subsequently, each sample was rinsed with phosphate buffered saline (PBS) for 6 times and dried at room temperature. Each neat and GO modified titanate nanowire scaffold was immersed in 500 μL culture medium containing MG63 cells and cultured in a humidified incubator with 5% CO_2_ at 37 °C. The culture medium was refreshed every two days.

### Fluorescence Microscopy of Cell Morphology

When MG63 cells were cultured for 2, 4 and 6 days, respectively, the culture medium with unattached cells was carefully removed from the culture wells by pipette, and then the samples were rinsed twice with PBS buffer. The cells on the samples were fixed with 2.5% glutaraldehyde in 0.1 M PBS buffer (pH = 7.4) for 20 min, and permeabilized 3 times with 0.1% Triton X-100 (Beyotime Institute of Biotechnology, China) diluted in PBS for 5 min at room temperature, respectively. For fluorescence imaging, the cytoskeleton of osteoblasts was stained with 500 mL Actin-Tracker Green (phalloidin-FITC, Beyotime Institute of Biotechnology, China; 30 mL phalloidin-FITC in 3 mL 0.1% Triton X-100 PBS) and the nucleus was counterstained with 4′-6-Diamidino-2-phenylindole DAPI (Beyotime Institute of Biotechnology, China; 5 mg/mL DAPI in MilliQ-water). Afterwards, the samples were photographed by a fluorescence microscope (BX51; ZEISS, Germany) with B- and G-light excited at 420 ~ 485 nm and 460 ~ 550 nm, respectively.

### Field Emission Scanning Electron Microscopy of Cell Morphology

When MG63 cells were cultured for 2, 4 and 6 days, the culture medium with unattached cells was carefully removed from the wells by pipette, and then rinsed 2 times with PBS buffer solution. The cells on the samples were fixed with 2.5% glutaraldehyde in 0.1 M PBS buffer (pH = 7.4) for 20 min, and washed 3 times with PBS for 5 min at room temperature. Afterwards, each sample was progressively dehydrated with 10%, 20%, 30%, 50%, 60%, 70%, 80%, 90% and absolutely ethanol for 10 min at room temperature, respectively, and then the samples were dried for SEM analysis.

### MTT and ALP Assay

After 2, 4 and 6 days of co-incubation, the culture medium was removed from culture wells, and the specimens were transferred to a new 24-cell culture plate. Subsequently, 100 μL of fresh culture medium and 20 μL of 3-(4, 5-dimethylthiazol-2-yl)-2, 5-diphenyl tetrazolium bromide (MTT, Sigma, USA)) solution (5 mg × mL^−1^) were added to each culture well for 4 h, and then the MTT solution was removed. Next, 200 μL of DMSO was added and ell viability was evaluated by using MTT reduction conversion assay and cell survival was expressed as absorbance at 570 nm in a spectrophotometric microplate reader (Bio-RAD Model 680, USA). The retention of the osteoblastic phenotype was examined by measuring the alkaline phosphatase (ALP) activity which based on the conversion of colorless p-nitrophenyl phosphate into colored p-nitrophenol (JianCheng Biotech., China). Color intensities were measured at 520 nm using the UV-vis spectrophotometer (DU530, Beckman Coulter, Inc., Fullerton, USA). In order to study the *in vitro* biocompatibility of the scaffolds, incubation of cells with the 1% triton (induce cell death) containing medium was considered as positive controls, whereas negative controls refer to incubation of cells with the medium. All data were based on optical density measurements. The results of MTT and ALP assay results were presented in the following sections.

### ARS Staining

Alizarin red-S (ARS) dye was utilized to identify calcium deposition given its high affinity to calcium salts selectively. The cell-scaffold constructs were rinsed twice with PBS, fixed in 70% ice-cold ethanol for 1 h, washed three times with deionized water and stained with 40 mM ARS for 30 min. After rinsing with deionized water, cells were detached from the constructs using 10% cetyl pyridinium chloride (CPC) for 1 h and measured for absorbance at 492 nm on a spectrophotometer (Thermo Spectronic, USA). All data were based on optical density measurements. The results of ARS staining were illustrated in the following sections.

### Protein Adsorption Examination

Protein adsorption was characterized to elucidate cellular response to the scaffolds that were incubated in five different concentrations of bovine serum albumin (BSA) solutions of 5 mL at room temperature for up to 12 h under static conditions. After immersed scaffolds were removed, the protein left in the medium was detected using a protein assay kit (DC protein assay kit, BioRad, USA). The data were normalized to a standard curve obtained with bovine serum albumin in the range of 0.012–0.5 mg/mL.

### Statistical Analysis

All data reported herein were a mean value of 6 replicates and were presented as mean values ± standard deviations (SD). Statistical analysis was performed using statistical software Origin 8.0. Significant differences between discrete groups measured using a *t*-test and *p*-values, were considered statistically significant at *p* < 0.05 and annotated with asterisks.

## Additional Information

**How to cite this article**: Dong, W. *et al.* A Dual Role of Graphene Oxide Sheet Deposition on Titanate Nanowire Scaffolds for Osteo-implantation: Mechanical Hardener and Surface Activity Regulator. *Sci. Rep.* **5**, 18266; doi: 10.1038/srep18266 (2015).

## Supplementary Material

Supplementary Information
