# Supplementary material for: A Dual Role of Graphene Oxide Sheet Deposition on Titanate Nanowire Scaffolds for Osteo-implantation: Mechanical Hardener and Surface Activity Regulator
Source: Sci Rep. 2015 Dec 21;5:18266. doi: 10.1038/srep18266 (PMC4685306; doi:10.1038/srep18266)
Supplement: Supplementary Information [file srep18266-s1.doc]

**Supplementary Information**

A Dual Role of Graphene Oxide Sheet Deposition on Titanate Nanowire Scaffolds for Osteo-implantation: Mechanical Hardener and Surface Activity Regulator

Wenjun Dong*^ab^, Lijuan Hou^a^, Tingting Li^a^, Ziqiang Gong^a^, Huandi Huang^a^, Ge Wang*^b^, Xiaobo Chen^c^& Xiaoyun Li^a^

^a^ Center for Nanoscience and Nanotechnology, Zhejiang Sci-Tech University, Hangzhou 310018, PR China

^b^School of Materials Science and Engineering, University of Science and Technology Beijing, Beijing 100083, PR China

^c^Department of Materials Science and Engineering, Monash University, Clayton, VIC. 3800, Australia

All correspondence should go to: Wenjun Dong (wdong@ustb.edu.cn) and Ge Wang (gewang@mater.ustb.edu.cn)


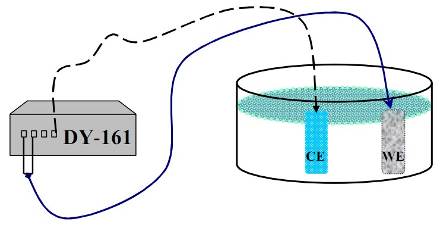


**Fig S1.** Schematic illustration of the setup of the electrophoretic deposition actuator, which was used to prepare GO sheets deposition onto titanate scaffolds.

**Spring table**

**Sample**

**Stage**


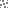

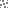

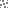


**Fig S2.** Schematic illustration of the setup for mechanical property measurement, performed inside the SEM chamber.


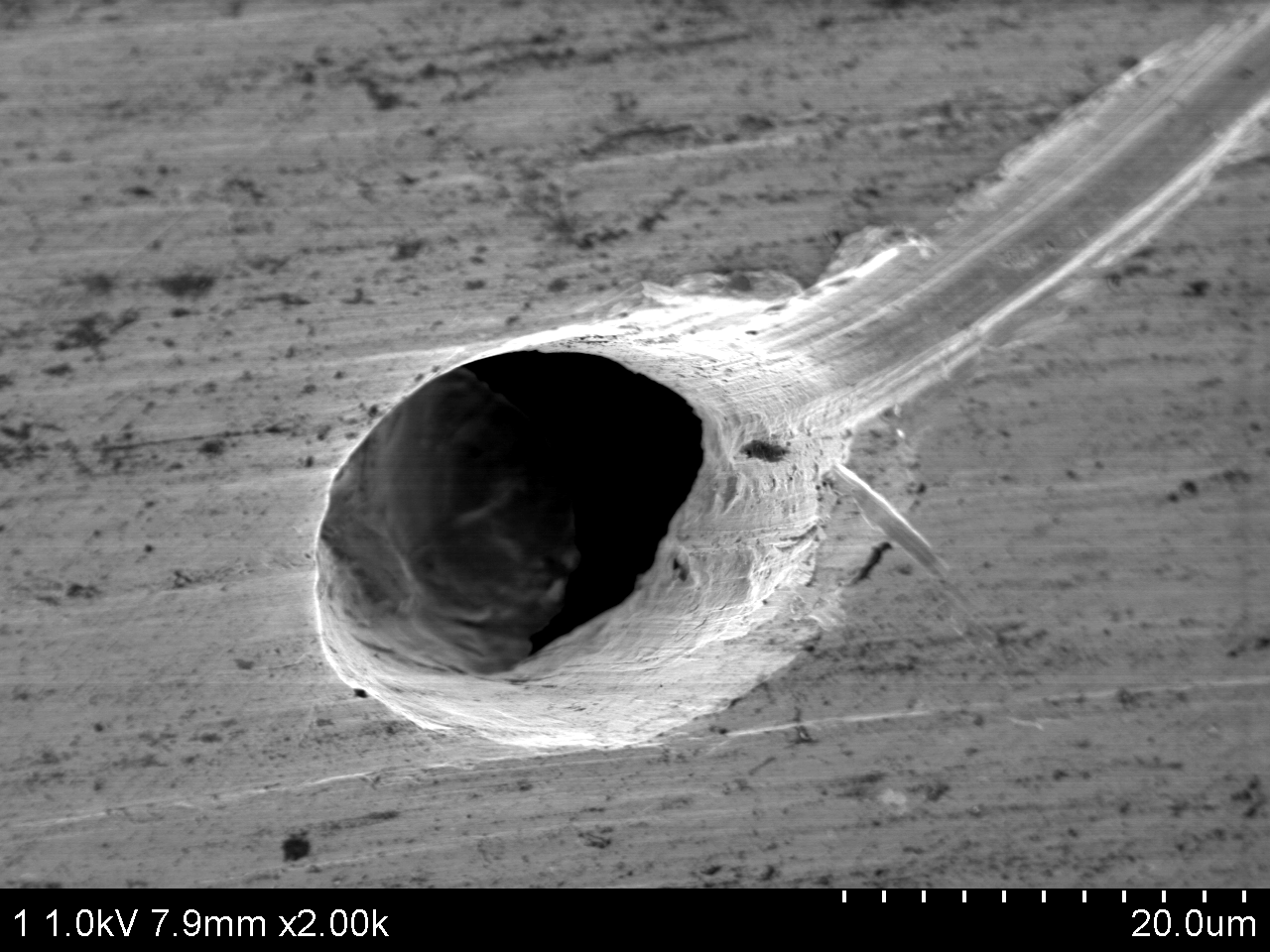


**D=1.12 µm**

**A**


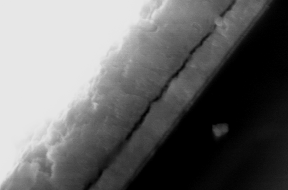


**L =14.76 μm**

**Fig S3.** SEM micrographs of (A) a scratch created by the needle scratched on aluminum foil. The diameter of the needle was measured at the innermost region, and (B) cross-section of porous titanante scaffold with a thickness of 14.76 μm residing on the surface of Ti substrate.

**B**


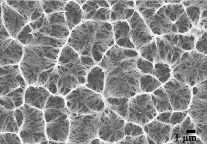

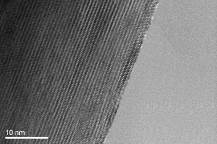


A

C

D

B

**Fig S4.** (A) SEM image, (B) HRTEM image and (C) XRD pattern of the neat titanate scaffold, and D) XRD pattern comparison of neat titanate and GO/titanate scaffold.


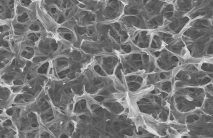

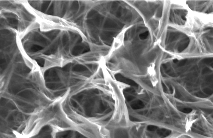


**C**

**C***


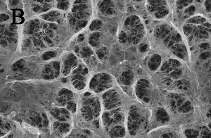

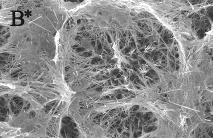


**B**

**B***


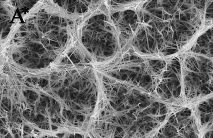

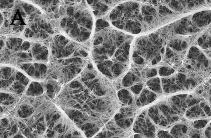


**A***

**A**

**20 µm**

**2 µm**

**20 µm**

**2 µm**

**20 µm**

**2 µm**

**Fig S5.** Surface morphology of GO modified titanate scaffolds electrodeposited in electrolytes with different pH: (A and A*) 7.2, (B and B*) 7.6 and (C and C*) 8.5, respectively, (Electrophoretic deposition condition: E*_appl_* =1.3 V and t = 5 min). It is evident that the quantity and density of the GO deposition on titanate scaffolds increases as a function of pH of electrolyte.

**Fig S6.** Surface morphology of GO modified titanate scaffolds electrodeposited at different potentials: (A and A*) 1.0, (B and B*) 1.2 and (C and C*) 1.3, respectively, (Electrophoretic deposition condition: pH = 8.5 and t = 5 min). It can conclude that 1.3 V_SCE_ is the premium condition to generate GO sheet deposition with uniform distribution and sufficient quantity and density to perform desired function.


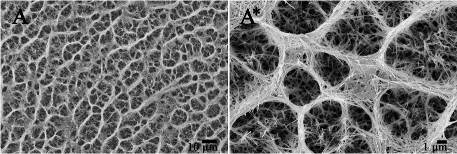

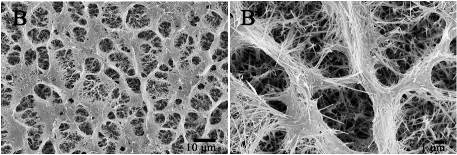

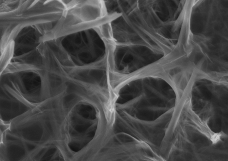

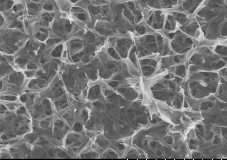


**20 µm**

**20 µm**

**20 µm**

**2 µm**

**2 µm**

**2 µm**

**B**

**C**

**B***

**C***

**A**

**A***

**Fig S7.** Plots of applied force as a function of indentation depth of various functionalized GO/titanate scaffolds that were fabricated through hydrothermal process for 4 h, indicating a comparative mechanical property to that of the GO/titanate scaffolds without functional terminals.


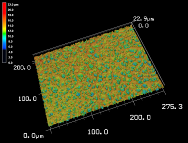

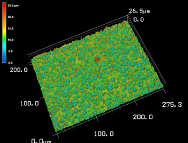

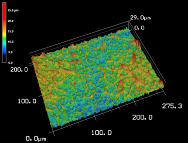

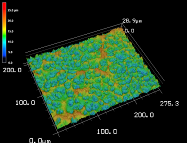

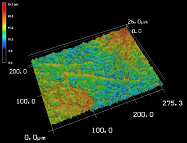


**a**

**b**

**c**

**d**

**e**

**Fig S8.** CLSM images and R_a_ values of (a) neat titanate scaffold, R_a_ = 1.80 µm; (b) GO/titanate scaffold, R_a_ = 1.94 µm; (c) -COOH grafted GO/titanate scaffold, R_a_ = 2.15 µm; (d) -OH grafted GO/titanate scaffold, R_a_ = 2.26 µm; and (e) -NH_2_ grafted GO/titanate scaffold, R_a_ = 2.16 µm. (Error bars represent standard deviation).

**Fig S9.** Protein adsorption on neat titanate scaffold, GO/titanate scaffold, -COOH grafted GO/titanate scaffold, -OH grafted GO/titanate scaffold, and -NH_2_ grafted GO/titanate scaffold after cultured in five different protein concentrations. (*Significant against adsorption on neat titanate scaffold at *p* < 0.05, and error bars represent standard deviation).
